# Rural Education

**Published:** 1903-02-07

**Authors:** 


The Hospital.

A Journal of

The Medical Sciences and Hospital Administration.

Vol. XXXIII.—No. 854. Edited by Sir Henry Burdett, K.C.B., and Solomon C. Smith, M.D., M.R.C.P. [February 7, 1903.

### Rural Education.

The Duke of Devonshire, who possesses in a high degree the inestimable gift of being able, in a few words, to reduce an apparently complicated question to its simplest terms, and to place it as a plain issue before an audience, has lately been exercising this gift at Derby, in order to instruct the dairy farmers of the county concerning their new relations to the conduct of national education. He told them, in effect, that in the future it must rest with themselves to see that the children of the county, in so far as they were educated at the county cost, were being educated in such a manner as to fit them for the work in life which, in all probability, they would be called upon to perform; and the lesson which he endeavoured to drive home is one which should be learnt, not by farmers only, but by many other classes of the community, with special inclusion of the medical profession. We have already insisted upon the great services which, if they be so minded, medical practitioners in rural districts may render in relation to the management of schools, not only in insisting upon the observance of sound physiological principles in the mere conduct of teaching, but also in taking care that the subjects taught are of such a nature as to afford useful guidance in the conduct of life. No one who has studied Miss Alice Ravenhill's reports upon schools in the United States with the attention which they deserve can entertain a doubt as to the extent to which these schools surpass our own in the direction of inculcating, not only sound sanitary principles, but their daily application to the preservation of health and the maintenance of cleanliness. The chief defect to which she calls attention in this matter is that an association of female total abstainers, by some of the "lobbying" or "log-rolling" processes known to American legislators, has been enabled to require of school managers that a considerable portion of the time supposed to be allotted to physiology shall be consumed in teaching the injurious effects of alcoholic drinks, and in doing this by the aid of text-books composed for and approved by the association, but of a character which does not commend itself to scientific people. On this ground alone, it is said, the teaching of physiology is to a great extent evaded, because those who are responsible for its conduct are unable to approve the books which are put into their hands, and have no power to reject them as unsuitable. Even when thus limited, the teaching is probably better than that which called forth, from a member of the elementary physiology class of an English Board school, the well-known account of the great cavities of the body and of their contents. "The cavities of the body are three in number: the head, which contains the brains, when there are any; the chest, which contains the heart and lungs; and the belly, which contains the vowels, which are a, e, i, o, u, and y."

Up to the present time, as the Duke told his Derbyshire audience, the education given in our Board schools has been far too exclusively of a so-called "literary" character, and has neither itself borne any sufficient relation to the facts of life, nor been supplemented by other methods of acquiring trustworthy information concerning them. The time then would seem to have come for a recognition of the fact that there are many instruments of teaching which are better than books. The "literary" education, as the Duke politely called it, serves chiefly to develop tastes and inclinations which can only be gratified in urban communities, and it thus becomes an important factor in that "exodus" from the country to the towns which it is impossible not to recognise and which it is customary to deplore. The education of the country should be largely based upon country topics, and should be planned in relation to country facilities and to the "sweetness" of "the lore that Nature brings." The scholars should be encouraged to devote their half-holidays to a naturalists' field club, to collect insects, to acquire a knowledge of their metamorphoses, and of the good or harm which they may do to crops. The operations of the farm, of the field, and of the garden should furnish material for lessons under which these operations would be transformed from objectless drudgery to intelligent co-operation with Nature. Even from the "literary" point of view, the time so spent would not be lost. Forty years ago the late Mr. Paget, M.P., established in the village school at Ruddington a system by which half the children were taken out of the class-rooms and employed in a garden for half their allotted school time. These children far outstripped the rest at the ordinary tasks of school, and the same thing would happen if the experiment were repeated. If the farmers are to carry out the Duke's principle, they will want the aid of the doctors; and, as we have said more than once, the Education Act has opened to the medical profession opportunities of public usefulness such as it has never before possessed. We can only hope that it will rise to and accept them.

